# The plant cuticle: old challenges, new perspectives

**DOI:** 10.1093/jxb/erx389

**Published:** 2017-11-08

**Authors:** Eva Domínguez, José A Heredia-Guerrero, Antonio Heredia

**Affiliations:** 1 Instituto de Hortofruticultura Subtropical y Mediterránea (IHSM) La Mayora. Universidad de Málaga-CSIC, Algarrobo-Costa, Málaga, Spain; 2 Smart Materials, Istituto Italiano di Tecnologia, Via Morego, Genova, Italy; 3 IHSM-CSIC-UMA, Departamento de Bioquímica y Biología Molecular, Facultad de Ciencias, Universidad de Málaga, Campus de Teatinos, Málaga, Spain

**Keywords:** Bioplastics, cell wall, cuticle, cutin, epidermal cells, epidermis, leaf surface, phyllosphere, suberin, transpiration, wax

**As the interface between plant and environment, the plant cuticle plays a significant number of roles many of which relate to protection – against dehydration, UV and other abiotic stresses such as heavy wind and rain, and moreover against the array of insects, fungi and other organisms which would do significant harm. The cuticle is thus important in terms of basic research, but also in crop protection and quality improvement. Its unique properties have also led to novel developments such as bioplastics. This special issue provides an overview of recent progress in this field, an interdisciplinary view of the cuticle that will stimulate future research.**

*What weave is this*

*of will be, is, and was?*

J. L. Borges

A review of the first book published on the plant cuticle almost 50 years ago (Martin and Juniper, 1970) stated how neglected the outer layers of epidermal cells had been in the plant biology literature, and expressed hope that the timely book would help in correcting this underestimation. These expectations have been greatly exceeded, and during the past two decades especially the amount of cuticle-related research has increased dramatically. These contributions range from plant taxonomy to polymer chemistry, including molecular biology, genetics, biophysics, biochemistry, plant pathology, ecophysiology, agronomy and plant evolution, among many others. Box 1 provides an overview of the way our understanding of the ‘plant cuticle concept’ has developed over the years.

The main function ascribed to the plant cuticle is protection against a dehydrating environment and UV radiation. But what of its origins? Unfortunately, its evolution is still poorly understood partly due to the limited number of species studied, even more so if we consider how little research has focused on the cuticle of non-spermatophytes (reviewed in Jeffree, 2006). Recently, starting to correct this imbalance, studies of the moss *Physcomitrella patens* have shown a cuticle enriched in phenolic compounds that seem to be a limiting factor in cuticle deposition (Buda *et al.*, 2013; Renault *et al.*, 2017). In this issue, Niklas *et al.* (2017) review phylogenetic and molecular analyses showing that the metabolic pathways involved in the biosynthesis of the monomers of cuticle and other related biopolymers such as suberin are ancient, and how the evolution of these pathways contributed to the early evolution of land plants.

Other state of the art reviews in the issue provide coverage across the subject, including emerging techniques and approaches. Some of the topics might be considered to focus on ‘old questions’ that are still not fully answered whereas others have arisen more recently from our evolving recognition and comprehension of the role of cuticle. Given the extraordinarily broad nature of the subject, other recent reviews in related areas, such as biophysics, cutin synthesis and postharvest, complement the contents (see Domínguez *et al.*, 2011b, 2015; Hen-Avivi *et al.*, 2014; Lara *et al.*, 2014; Martin and Rose, 2014; Fernández *et al.*, 2016; Fich *et al.*, 2016).

## Survival in a dehydrating environment

In order to survive in a dehydrating environment, plants need to maintain an equilibrium between water loss and root water uptake. Transpiration is controlled by the stomata and the cuticle, and although stomatal conductance is a few orders of magnitude higher than cuticle conductance, water status under conditions of stomatal closure depends on the rate of cuticle transpiration (Kerstiens, 1996). Due to its physiological relevance, cuticle transpiration has been amply studied in a significant range of species and environmental conditions, and different models to explain water movement across the cuticle have been postulated (Burghardt and Riederer, 2006; Kerstiens *et al.*, 2006; Schreiber and Schönherr, 2009). Considering the variety of adaptations and specialized structures present in different species, it is important to address, in an ecophysiological context, the role of cuticle transpiration in comparison to other strategies employed by plants to minimize water loss. From this viewpoint, Schuster *et al.* (2017) review the topic of leaf cuticle transpiration in a large number of plant species, questioning the commonly accepted notion of a direct relationship between cuticle water permeability and abiotic ecological factors.

Several methodologies have been employed to measure cuticle transpiration, from using isolated astomatous or hypostomatous cuticles to indirect protocols such as chlorophyll extraction and Toluidine O staining to assess cuticle permeability in detached leaves of species in which the cuticle cannot be isolated (Lolle *et al.*, 1997; Tanaka *et al.*, 2004). This variability in technique suggests that it might be difficult to compare results from different sources – each may give a different estimate. Zeisler-Diehl *et al.* (2017) critically review methodologies employed to study and analyze cuticle barrier properties, emphasizing their different requirements and problems.

**Box. 1. Evolution of the plant cuticle concept**

Diagrams taken from the literature show the evolution of the plant cuticle concept, and how our understanding and interpretation of the available data have changed with time. Early views of the cuticle identified it as an extracellular lipid layer deposited on top of, but not connected to, the epidermal cell walls (upper left; Martens, 1933); later on the presence of a cellulosic (polysaccharide) domain was acknowledged and therefore an interconnection with the outer epidermal cell walls (upper right, showing the orientation of cellulose, cutin and wax; Meyer, 1937). Additionally, changes in the cuticle during organ growth and development were identified, from a thin and mainly lipid layer to a more complex structure in mature organs (lower left, showing phases of cuticular development; Sargent, 1976). Reproduced, with permission, from *Annals of Botany* (C. Sargent, The occurrence of a secondary cuticle in *Libertia elegans* (Iridaceae), *Annals of Botany*, 1976, vol. 40, pp. 355–359). Our current view of the cuticle is far more complex: it is described as a composite, anisotropic and heterogeneously distributed biopolymer (Jeffree, 2006). The lower right diagram shows this complexity: CL, cuticle layer; CP, cuticle proper; CYS, cutin cystoliths; ECL, external cuticle layer; EWC, epicuticular wax crystals; EWF, epicuticular wax film; ICL, internal cuticle layer; SCW, secondary cell wall (Jeffree, 2006). Reproduced, with permission, from John Wiley and Sons (C.E. Jeffree, The fine structure of the plant cuticle, in M. Riederer, C. Müller, eds, *Biology of the plant cuticle*, 2006. Oxford: Blackwell, pp. 11–125).

**Figure:**
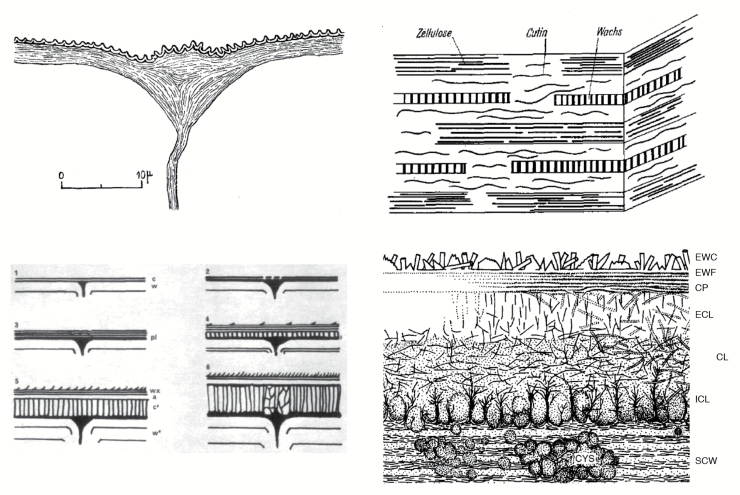

Given the composite nature of the cuticle, determination of the participation of each component to water permeability is an important issue. The chemical nature of waxes, mainly the amount of intracuticular very long chain aliphatics, has been identified as the main barrier to water movement across the cuticle (Vogg *et al.*, 2004), although recent studies suggest a more complex scenario with epicuticular waxes contributing to permeability in some cases (Jetter and Riederer, 2016). Hence, the chemical properties of cuticle and the distribution of cuticle components are of great importance in understanding surface wettability and water movement. Fernández *et al.* (2017) address this topic, studying the physico-chemical properties of the cuticle and its role as a barrier to water and electrolyte deposition and absorption. The authors conclude that the significant structural and chemical variability exhibited by plants prevent the establishment of general models predicting liquid–cuticle surface interactions.

## Complex interactions revealed with model plants

The use of Arabidopsis as a plant model for cuticle studies, and more recently of tomato, has allowed the identification of numerous genes involved in cuticle synthesis and deposition. It has also prompted the recognition of complex interactions between pathways that were not previously known to be connected. In this regard, the initial work of Lolle *et al.* (1992) uncovered a role of the cuticle as a barrier to the transport of signals participating in cell adhesion between organs in close proximity, thus preventing organ fusion. Moreover, a relationship between epidermal cell identity or cell size and cuticle development has been observed in tomato (Nadakuduti *et al.*, 2012; España *et al.*, 2014). Very long chain fatty acids, one of the main components of cuticle waxes, have been implicated in recent years as signal molecules intervening in several processes related to epidermal cell division and expansion and defence mechanisms (Raffaele *et al.*, 2009; Nobusawa *et al.*, 2013). In their review, Ingram and Nawrath (2017) discuss the basis of developmental phenotypes associated with defects in cuticle function and the mechanisms underlying developmental processes that implicate cuticle modification.

## Resisting other organisms and mechanical properties

The phyllosphere is the physically and chemically distinct surface environment where insects, fungi, yeast and bacteria interact with the plant. Insects will explore the plant surface searching for food, a place for oviposition or shelter. Plants show different strategies to prevent insect colonization, from the synthesis of deterrent compounds to impeding insect attachment. Gorb and Gorb (2017) examine plant cuticle adaptations, especially the three-dimensional structure of epicuticular waxes, related to the prevention of insect attachment. They also discuss current hypotheses that explain insect–wax interactions and their ecological implications. The role of the cuticle in plant interaction with pathogens has benefited from the work carried out with Arabidopsis mutants displaying cuticle defects. Such mutants have allowed the identification of some cuticle components participating in the activation of the plant immune response. Aragón *et al.* (2017) focus on the interaction between microorganisms and the leaf surface and review the role of cuticle components in the complex processes of pre-invasion, infection and further plant responses.

The cuticle mechanically protects plants by reducing the impact of external stresses such as wind or heavy rain and, in conjunction with the epidermis, preventing tissue breaking and participating in the control of organ growth (Savaldi-Goldstein *et al.*, 2007; Dominguez *et al.*, 2011a). Over the past decade, the physiological and agronomic implications of the mechanical properties of plant cuticles have raised a lot of interest, from their involvement in fruit cracking, a disorder that causes severe economic losses in many fleshy fruits, to their complex role in fungal growth. It has been reported that, whereas a stiff surface can increase the physical force needed for fungal penetration, a soft surface abrasion could induce resistance to fungal growth (Beniklhef *et al.*, 2013; Ryder and Talbot, 2015; Aragón *et al.*, 2017). Plant biomechanics is a subject that involves basic physics in order to identify the stresses that a plant is subjected to and the development of the appropriate mechanical test. In this issue, Khanal and Knoche (2017) extensively review the mechanical behaviour of plant cuticles and epidermises, with special emphasis on the experimental conditions needed to measure mechanical parameters and their physical and physiological relevance.

## Crop improvement to bioplastics

The importance of the cuticle in many traits related to plant growth/performance and fruit quality has become more apparent in the past decade and has driven a significant amount of research on crop improvement focused on cuticle characteristics (Domínguez *et al.*, 2012; Domínguez and Heredia, 2017). The review by Petit *et al*. (2017) examines cuticle traits of agronomic importance, with a special focus on fleshy fruits. The review also discusses the usefulness of modern strategies such as genome-wide association studies (GWAS) and CRISPR–Cas9 gene-editing for exploiting natural and artificially induced genetic variability of cuticle-associated traits for breeding purposes. Additionally, the agronomic implications and potential application of cuticle biomechanics, movement of solutes across the cuticle and cuticle participation in pest control have been considered by Aragón *et al.* (2017), Fernández *et al.* (2017) and Khanal and Knoche (2017).

Molecular assembly of the different building blocks responsible for the final architecture of the plant cuticle is one of the most intriguing current lines of research. In spite of the significant progress that has been made in recent years, we are still far from understanding the mechanisms that take place in the outer epidermal cells (Domínguez *et al.*, 2015; Fich *et al.*, 2016). In their comprehensive review, Cohen *et al.* (2017) summarize the latest results on the identification of genes and developmental programmes involved in cuticle and suberin biosynthesis using large-scale strategies and other novel technologies such as mass spectrometry imaging (MSI).

For many years, polymer scientists have looked at nature for inspiration. Natural biopolymers combine a series of intrinsic characteristics with the ability to adapt them to environmental changes that are of great interest in this applied field. The plant cuticle exhibits a combination of barrier properties such as reduced water loss and protection against UV radiation and pathogen attack together with a high biodegradability and non-toxicity that has attracted the attention of researchers to create a bio-inspired synthetic replica as a substitute for petroleum-based plastics. The initial work of Benítez *et al.* (2004) showed the potential use of cutin monomers for developing bioplastics with special characteristics, prompting their revalorization as raw material. In this issue, Heredia-Guerrero *et al.* (2017) provide a state of the art summary of the fabrication of cutin-based materials. Furthermore, the main characteristics of cutin are compared with those of common plastics, together with potential sources and annual biomass.

## Perspectives: have I asked the right question?

As already mentioned, there is an imbalance between the number of plant species studied at the cuticle level and the number of extant species. Moreover, very few non-vascular plants or vascular plants other than angiosperms have been analyzed. This has limited study of the evolution of the cuticle or the potential identification of phylogenetic branches in which plants share similar cuticle composition, structure and properties. Cuticle studies have mainly been limited to species whose cuticles can be isolated. These allow an analysis of the properties of the isolated cuticle and comparison with the cuticle in the intact tissue, organ or plant. However, many cuticles are too delicate to be isolated and so approaches have been developed to analyze them indirectly, although at the risk of contamination from inner tissues that could lead to misinterpretation (Schreiber and Schönherr, 2009). In early stages of development and in some green algae, cuticle detection is only based on the presence of a nanoscopic osmiophilic layer, with no information pertaining to its chemical composition (Jeffree, 2006). Nevertheless, in recent years there have been tremendous advances in microscopy allowing, for example, the spatial resolution of electron microscopy to be combined with spectroscopy, electron crystallography or tomography (Kourkoutis *et al.*, 2012). Development and application of such techniques to biological samples will provide a methodology for identifying cuticles *in situ* and additional information about the structure, heterogeneous distribution of components and location of certain ions.

The last decade has been characterized by a multidisciplinary research approach that has uncovered connections between different developmental processes such as cuticle deposition and environmental conditions, cuticle synthesis and epidermal identity or cell expansion, and mechanical properties (of the cuticle) and cell expansion, to name just a few (Cohen *et al.*, 2017; Ingram and Nawrath, 2017; Khanal and Knoche, 2017). In order to fully understand these interactions it will be necessary to integrate existing knowledge of the cuticle within the context of the epidermal tissue, the organ (e.g. leaf, stem, fruit) and, moreover, the whole plant. This could help identify strategies employed by different plant species to achieve similar goals. For example, the lack of correlation between cuticle thickness or permeability and environmental habitat could be explained by different species developing alternative strategies (Schuster *et al.*, 2017). In a similar fashion, in some species the mechanical resistance of the skin is mainly sustained by the epidermal cell walls with little contribution of the cuticle (Khanal and Knoche, 2017).

Our understanding of the plant cuticle has evolved significantly over the nearly 50 years since Martin and Juniper’s seminal book, mentioned at the beginning, and has revealed a significant level of complexity. This complexity should encourage us to discover new questions that will open new research paths. Scientific enquiry is sustained by the heuristic power of a question that is continuously being refined as more information and knowledge is accumulated. With this philosophy, we believe that a multidisciplinary methodology, combining diverse areas, should still be pursued and will provide a better understanding of this biopolymer. And, if at some point things get too complicated, remember the words of mathematician Enrico Bombieri, and ponder: have I asked the right question?
